# Are statins making older persons weaker? A discontinuation study of muscular side effects

**DOI:** 10.1007/s11357-023-00817-2

**Published:** 2023-05-25

**Authors:** Morten Bruun Korsholm, Thea Winther Pødenphanth, Sofie Kirstine Strømgaard, Linda Wiuff Petersen, Christina Alexandersen, Sarah Samama Hoffmann, Hanne K. Rasmusen, Charlotte Suetta, Kirsten Damgaard, Eckart Pressel, Flemming Dela

**Affiliations:** 1 Department of Geriatric and Palliative Medicine, Copenhagen University Hospital - Bispebjerg and Frederiksberg, Copenhagen, NV Denmark; 2 Department of Medicine, Copenhagen University Hospital Herlev-Gentofte, Herlev, Denmark; 3 Department of Neurology and Geriatrics, Copenhagen University Hospital - Næstved, Slagelse and Ringsted, Slagelse, Denmark; 4 Xlab, Center for Healthy Aging, Department of Biomedical Sciences, Faculty of Health and Medical Sciences, University of Copenhagen, 2200 Copenhagen, Denmark; 5 Department of Cardiology, Copenhagen University Hospital - Bispebjerg and Frederiksberg, Copenhagen, Denmark

**Keywords:** Myalgia, Muscle performance, Statins

## Abstract

Thirteen percent of the Danish population are treated with a statin—half of these are in primary prevention, and most are > 65 years old. Statins have known muscular side effects (i.e., myalgia) correlated to reduced muscle performance. This study examines if years of statin treatment in older people introduce subclinical muscle discomfort and loss of muscle mass and strength. In total, 98 participants (71.1 ± 3.6 years (mean ± SD)), who were in primary prevention treatment for elevated plasma cholesterol with a statin, were included in this study. Statin treatment was discontinued for 2 months and then re-introduced for 2 months. Primary outcomes included muscle performance and myalgia. Secondary outcomes included lean mass and plasma cholesterol. Functional muscle capacity measured as a 6-min walk test increased after discontinuation (from 542 ± 88 to 555 ± 91 m, *P* < 0.05) and remained increased after re-introduction (557 ± 94 m). Similar significant results were found with a chair stand test (15.7 ± 4.3 to 16.3 ± 4.9 repetitions/30 s) and a quadriceps muscle test. Muscle discomfort during rest did not change significantly with discontinuation (visual analog scale from 0.9 ± 1.7 to 0.6 ± 1.4) but increased (*P* < 0.05) with the re-introduction (to 1.2 ± 2.0) and muscle discomfort during activity decreased (*P* < 0.05) with discontinuation (from 2.5 ± 2.6 to 1.9 ± 2.3). After 2 weeks of discontinuation, low-density lipoprotein cholesterol increased from 2.2 ± 0.5 to 3.9 ± 0.8 mM and remained elevated until the re-introduction of statins (*P* < 0.05). Significant and lasting improvements in muscle performance and myalgia were found at the discontinuation and re-introduction of statins. The results indicate a possible statin-related loss of muscle performance in older persons that needs further examination.

## Introduction

Statins (HMG-CoA reductase inhibitors) are used worldwide to lower blood cholesterol [1]. Since their introduction 34 years ago, statins have become one of the most prescribed drugs in the Western world [2]. Globally, the number of patients taking a statin has increased by 4–5% annually over the last decade, with most statin users in Europe and North America [3].

In 2021, 740,755 Danes were treated with a statin, corresponding to about 13% of the population [4]. These numbers are comparable to other Nordic countries like Norway [5], but higher than other northern European countries like France and the UK where 8–10% of the population take statins [6, 7]. More than half of the statin-treated individuals in Western countries are in primary prevention and have no other major risk factors for cardiovascular disease [6, 8–10].

About 2/3 (64%) of statin-treated patients in Denmark are above 65 years of age [4, 11]. Among older patients, the continuation of statin treatment for primary prevention purposes is a matter of concern. We do not know if years of statin treatment in this group have contributed to an acceleration of the known age-related loss of muscle mass and strength, a loss that has significant implications for individual independence and ability to maintain activities of daily living.

The potentially negative effect on the muscle of older people could be due to side effects, of which myalgia (muscle pain or aching, stiffness, tenderness, or cramps) occurs in 3–21% of statin users [12–14]. Myalgia is a strong disincentive to regular exercise [15, 16]. In addition, statin treatment may also negatively affect metabolism and energy production capacity (mitochondrial dysfunction) [17, 18].

A large part of the European statin treatment guidelines are based on the SCORE2/SCORE2-OP algorithm [19, 20]. In these, most Western European countries are considered low (i.e., Denmark, Spain, France, and the UK) to moderate (i.e., Germany, Italy, and Sweden) risk countries in regard to a citizen’s lifetime risk of a severe cardiovascular event. The latest European Society of Cardiology (ESC) guidelines from 2021 [19] changed recommendations for primary prevention to a more age-differentiated recommendation. Using SCORE2-OP individuals aged 70 years and older with scores < 7.5% (10-year risk of a cardiovascular event) is now considered low-to-moderate risk (previously 5%), and strong statin consideration is now above 15% risk (previously 10%). This should result in fever in older persons in primary prevention compared with the 2019 ESC recommendations, where upwards of 90% of 70–79-year-olds fulfilled the requirements for primary prevention with a statin [21].

Some studies have shown that for older people morbidity but not mortality is reduced with statin treatment [22, 23]. However, large cohort studies are challenging this perception and reporting that all-cause mortality is lowered by statins and low-density lipoprotein cholesterol (LDL-C) reduction in all age groups [11, 24, 25]. Most agree that the absolute benefits (reduced morbidity compared to adverse effects) of statins are diminished with increasing age and non-cardiovascular comorbidity. Therefore, it is important to continually examine the prescription (and continuation) of statins in primary prevention of patients > 65 years.

The clinical decision to initiate, continue, or discontinue a preventive medication in older people is a complex decision to make. Considerations about possible side effects, polypharmacy, life expectancy, and medicalization are important issues for older people. These people are not ill—they are at potential risk for a disease that may appear 5–10–15 years later. In this context, it is noteworthy that functional muscle capacity has a marked impact on 10-year mortality risk in people > 60 years [26, 27]. Other studies have also found a significant positive correlation between physical activity and a reduction in mortality in older people [28, 29].

Previously, a study showed no differences in strength and aerobic exercise performance between statin users, with or without myalgia, and a control group [30], while another study has shown a decrease in muscle performance due to statin treatment [31]. Several studies show an average increase in creatinine kinase (CK) and myalgia, but no certain muscular dysfunction [32, 33].

Thus, it remains unclear to what extent statins have an impact on physical activity and muscle performance, particularly in older (> 65 years) people. Previous studies in the field have not studied this in relation to the discontinuation of statin treatment and subsequent re-introduction of statins, and hence the effects on the individual in primary prevention.

The main objective of this study was to investigate muscle performance changes and myalgia after 2 months of discontinuation of statin treatment. Secondary outcomes focused on changes in lean mass and blood lipid concentrations.

## Methods

### Study design

We have performed an interventional study where participants were used as their own control (within-subject design). It was conducted as a multicenter study with the recruitment of eligible participants for the medical departments of three of the major hospitals in eastern Denmark. The departments were the Department of Medicine, Copenhagen University Hospital—Herlev-Gentofte, Department of Geriatric and Palliative Medicine, Copenhagen University Hospital—Bispebjerg and Frederiksberg, and Department of Neurology and Geriatrics, Copenhagen University Hospital—Næstved, Slagelse, and Ringsted.

### Recruitment

**Fig. 1:**
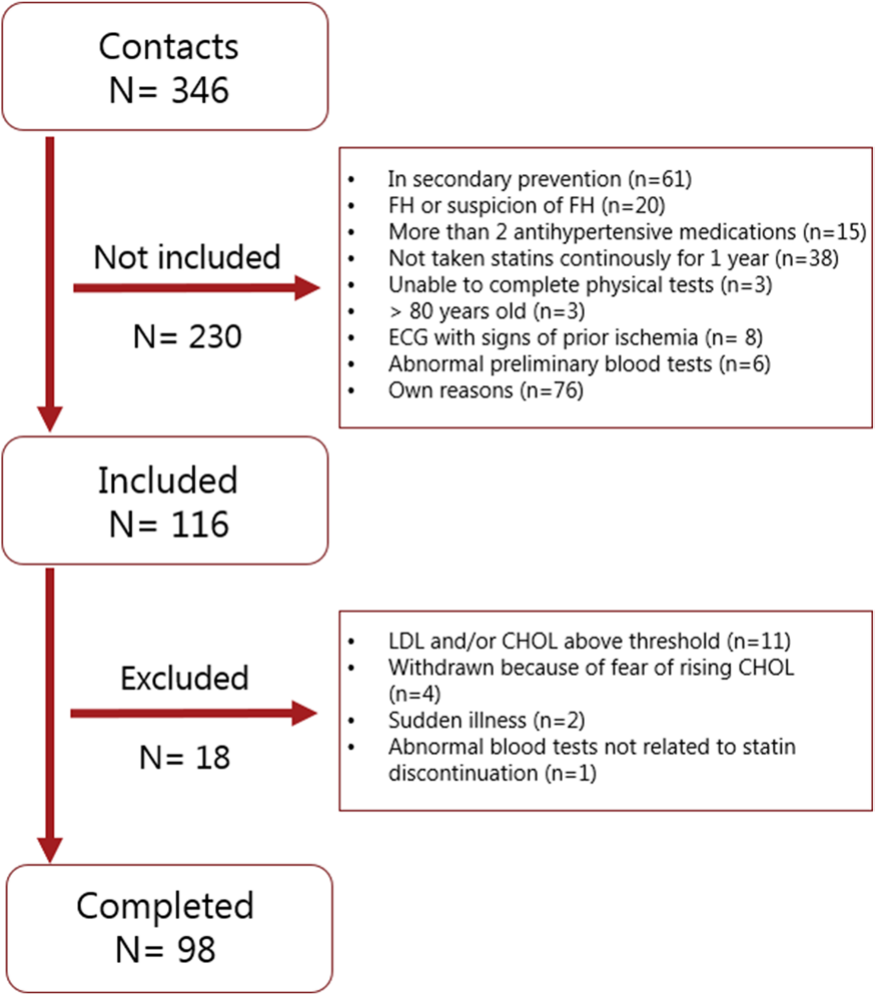
Consort diagram. Inclusion and exclusion criteria were set before the start of the study. Thresholds were set at 8.0 mM for plasma total cholesterol, 5.0 mM for low-density lipoprotein (LDL), and 5.0 mM for triglycerides (none was excluded because of triglycerides). ECG with signs of prior ischemia included left bundle branch block, negative T waves in adjacent leads, Q waves, or atrioventricular block of more than first degree

Enrollment was done via advertisement on social media where participants completed an electronic form and were thereafter contacted by phone for a brief inclusion interview (Fig. 1).

### Inclusion criteria

Inclusion criteria included men and women (65–80 years) in primary prevention treatment for elevated blood cholesterol and/or LDL-C with a statin (simvastatin, atorvastatin, rosuvastatin, pravastatin, etc.).

Participants had to have taken the same statin and dosage continuously and daily for a minimum of 1 year. Prior or present myalgia was not needed for inclusion.

### Exclusion criteria

Exclusion criteria included prior cardiovascular disease (minor-to-moderate hypertension was allowed, defined as treatment with maximally two different antihypertensive drugs), prescription of statin treatment due to familial hypercholesterolemia, type 2 diabetes, epilepsy, and kidney disease (glomerular filtration rate < 50 mL/min), and inability to complete the physical tests of the study.

Blood samples at baseline were required to be within the normal range if they could affect muscle function (e.g., thyroid-stimulating hormone and hemoglobin).

During the discontinuation phase, if total cholesterol or LDL-C rose above a preset level of 8.0 mmol/L or 5.0 mmol/L, respectively, the patients’ collective risk factors (blood pressure, smoking status, cholesterol, family history, etc.) were conferred with the study cardiologist who decided if the participant should be excluded based on the risk of familial hypercholesterolemia and/or a cardiovascular event.

### Intervention

**Fig. 2:**
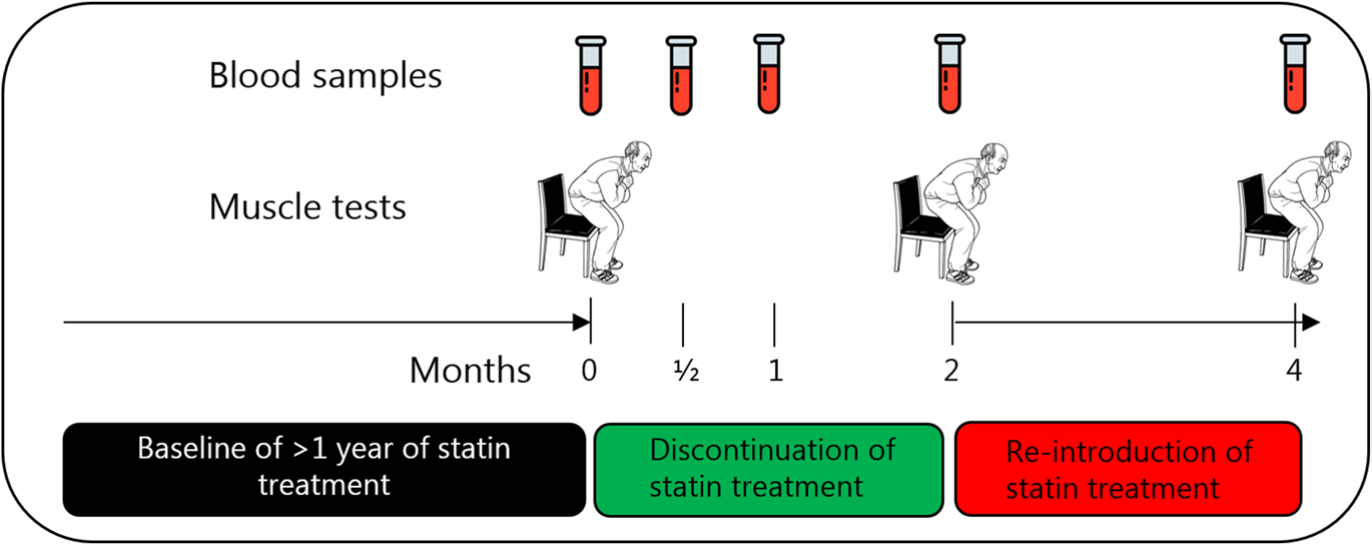
Study timeline. Participants in primary prevention with a statin were examined at baseline and then asked to discontinue statins for 2 months. During the 2-month statin discontinuation, plasma cholesterol was measured twice. Each participant was tested after 2 months of discontinuation and finally after 2 months of re-introduction of statins

The study included patients who discontinued their regular statin treatment for 2 months, where after the statin treatment was re-introduced (Fig. 2).

Participants were instructed to maintain their normal activities and lifestyle during the study.

Subject inclusion began on October 1, 2020, and was concluded by March 1, 2022. Inclusion was not affected by the corona pandemic apart from a few individuals who lost interest before inclusion because of social restrictions.

### Measurements

All tests were performed at baseline, after 2 months of discontinuation of the individual statin treatment and following 2 months of re-introduction.

#### Body composition

Weight, height, and calf and thigh circumference were measured.

Whole-body composition, i.e., lean mass, and body fat distribution were measured by DXA scan (Lunar iDXA, GE Medical Systems Lunar, USA).

#### Muscle strength

Handgrip strength (HGS): Muscle strength by a handgrip dynamometer (Jamar Smart Digital Hand dynamometer (PROcare, Roskilde, Denmark)) was measured. Participants received standardized instructions whereafter the best result out of three attempts was noted.

Quadriceps muscle test (QMT): Maximal isometric muscle strength of the quadriceps muscle was assessed with the participant in a seated position using a hand-held dynamometer (Lafayette) [34] fixated against a wall. This test only included a subgroup of the last measured participants, *N* = 19.

#### Functional muscle capacity

Six-minute walk test (6MWT): This test included walking as far as possible in 6 min between two cones on a 20-m track. Total distance and subsequent myalgia on a visual analog scale (VAS) scale were noted.

Chair stand test (CST): This test included 30 s of alternating between sitting on a chair and being in a fully standing position, and the number of repetitions was noted. Based on the weight and height of the participant, the number of repetitions was converted to muscle power and relative power (W/kg bodyweight) [35].

#### Physical activities score and presence of myalgia

The following two brief questionnaires were performed:

1. VAS measurement of muscle discomfort/myalgia [36]: Each participant was asked at each trial to rate their muscle discomfort in the legs and arms during the last 2 weeks. One answer for discomfort during rest and one during activity. The scale used was a physical Wong-Baker scale converted to a numerical value of 0.0–10.0 (VAS) [37].
2. International Physical Activities Questionnaire–Short Form (IPAQ-SF) [38]: A brief questionnaire on daily activity during the last 7 days. An IPAQ score of estimated recent physical activity was subsequently calculated.

#### Serologic markers

Blood samples were obtained on a total of five occasions for every participant, three times in relation to testing days, and two extra cholesterol blood samples, respectively, 2 and 4 weeks after discontinuation to follow the temporal changes in plasma cholesterol concentrations.

Besides total cholesterol, lipoproteins, and triglycerides, biochemical analysis consisted of Na^+^, K^+^, creatinine, creatine kinase, alanine aminotransferase, alkaline phosphatases, lactate dehydrogenase, International Normalized Ratio (coagulation factor II, VII, and X), platelets, C-reactive protein, C-peptide, hemoglobin, hemoglobin A1C, and thyroid-stimulating hormone (Table 1).

**Table 1:** Blood samples obtained at each of the three study visits

Mean ± SD	Baseline	2-month discontinuation	2-month re-introduction
Hemoglobin (mmol/L)	8.7 ± 0.6	8.8 ± 0.6	8.6 ± 0.7
Platelets (10^9^/L)	243 ± 46	250 ± 43	241 ± 47
Albumin (g/L)	39 ± 5	38 ± 3	39 ± 3
eGFR (mL/min/1.73 m^2^)	78 ± 12	77 ± 11	77 ± 14
Potassium (mmol/L)	4.0 ± 0.3	4.0 ± 0.3	4.0 ± 0.5
Creatinine (µmol/L)	73 ± 15	74 ± 14	74 ± 19
Sodium (mmol/L)	140 ± 2	139 ± 2	140 ± 2
Alanine aminotransferase (U/L)	27 ± 8	24 ± 9	29 ± 27
Alkaline phosphatase (U/L)	76 ± 18	77 ± 20	75 ± 19
Creatine kinase (U/L)	116 ± 73	106 ± 48	111 ± 54
Glucose (mmol/L)	5.9 ± 1.0	5.8 ± 0.9	5.6 ± 0.7
HbA1c (mmol/mmol)	37.9 ± 4.8	37.6 ± 3.3	38.0 ± 4.1
Thyrotropin (U × 10^−3^/L)	1.7 ± 1.0	1.7 ± 0.9	1.6 ± 0.9
C-peptide (pmol/L)	974 ± 594	873 ± 487	883 ± 551

Baseline, after 2 months of discontinuing statins, and after 2 months of re-introducing the same statin. Only plasma cholesterol, lipoproteins, and triglyceride (Fig. 4) were obtained more frequently. Biochemical analyses were chosen based on what could impact the muscle performances of the participants

### Statistics

Mixed effect analysis was used to analyze repeated measures (baseline ➔ discontinuation ➔ re-introduction) data (GraphPad Prism 9.0). This mixed model uses a compound symmetry covariance matrix and is fit using restricted maximum likelihood. In the absence of missing values, this method gives the same *P* values and multiple comparison tests as repeated measures ANOVA. In the presence of missing values (missing completely at random), the results can be interpreted as repeated measures ANOVA. *P* value < 0.05 was considered significant. In case of a significant main effect, Tukey’s multiple comparisons test was used as a post hoc test.

### Ethical considerations

The study was approved by the Danish ethics committee (H-20026737) and written informed consent was obtained from every participant.

## Results

### Baseline characteristics

The study group consisted of 98 individuals with a mean age of 71.1 ± 3.6 years, 72% women (Table 2). They were subcategorized based on their statin equipotent dosage: 34 participants took a low dosage (simvastatin 10–20 mg, atorvastatin 10 mg, and pravastatin 40 mg), 37 took a medium dosage (simvastatin 40 mg, atorvastatin 20 mg, and rosuvastatin 5 mg), and 27 were on a high dosage (simvastatin 60 mg, atorvastatin 40–80 mg, and rosuvastatin 10 mg). Equipotent dosage was based on the latest systematic review [32].

**Table 2:** Baseline characteristics

Age (yr)	71.1 ± 3.6
65–69 (yr)	42
70–74 (yr)	41
75–79 (yr)	15
Sex	
Female *n* (%)	71 (72.4)
Male *n* (%)	27 (27.6)
Body measures	
Height (cm)	166.5 ± 9.2
Body mass (kg)	74.5 ± 12.7
BMI (kg/m^2^)	26.8 ± 3.8
Duration of statin treatment (yr)	8.1 ± 5.7
1–4 yr (*n*)	35
5–9 yr (*n*)	21
10 + yr (*n*)	40
Unknown (> 1 yr; *n*)	2
Type of statin (*n*)	
Pravastatin	1
Simvastatin	32
Atorvastatin	56
Rosuvastatin	9
Equipotent statin dosage (*n*)	
Low (simvastatin 10–20 mg, atorvastatin 10 mg, and pravastatin 40 mg)	34
Medium (simvastatin 40 mg, atorvastatin 20 mg, and rosuvastatin 5 mg)	37
High (simvastatin 60 mg, atorvastatin 40–80 mg, and rosuvastatin 10 mg)	27
Medications per participant (average *number of prescribed drugs*)	3.3
Cardiovascular (i.e., antihypertensive and statins)	1.8
Pain- and musculoskeletal medication	0.4
CNS medication (i.e., SSRI)	0.1
Metabolic medication (i.e., levothyroxine)	0.1
Respiratory medication	0.2
Other	0.8
Hypertension (*n*)	
Registered hypertension	55
No registered hypertension	43
Smoking status (*n*)	
Active smoker	10
Non-smoker or previous smoker	88

Most participants were female and around 70 years old. About half of the participants had hypertension, which made antihypertensive medication the most used medication type after statins

Test participants had a BMI of 26.8 ± 3.8 kg/m^2^ with an average body fat of 37.6 ± 7.2 kg and an average total lean mass of 44.4 ± 8.2 kg (Table 3).

**Table 3:** Overall study findings

	Baseline	2-month discontinuation	2-month re-introduction
Mean ± SD			
Body composition			
Body mass (kg)	74.5 ± 12.7	74.2 ± 12.5	74.2 ± 12.5
BMI (kg/m^2^)	26.8 ± 3.8	26.6 ± 3.8	26.8 ± 3.9
Body fat (%)	37.6 ± 7.2	37.8 ± 7.1	37.6 ± 7.0
Thigh circumference average (cm)	50.5 ± 4.5	50.3 ± 4.8	50.0 ± 5.1
Calf circumference average (cm)	36.8 ± 3.1	37.1 ± 3.6	37.7 ± 4.3
Lean mass, total (kg)	44.4 ± 8.2	44.1 ± 8.1	44.3 ± 8.1
Lean mass, legs (kg)	15.2 ± 3.2	15.0 ± 3.2	15.1 ± 3.2
Lean mass, arms (kg)	4.8 ± 1.4	4.8 ± 1.3	4.9 ± 1.4
Physical function			
CST (#rep/30 s)	15.7 ± 4.3	16.3* ± 4.9	16.4* ± 4.9
Power (W)	268 ± 100	276* ± 102	278* ± 103
Relative power (W/kg)	3.6 ± 1.1	3.7 ± 1.2	3.7 ± 1.2
6MWT (m)	544 ± 78	556* ± 80	563* ± 90
Muscle strength test			
Hand dynamometer (kg)	31 ± 8	31 ± 9	31 ± 8
Quadriceps muscle test (kg)	120 ± 28	132* ± 35	139* ± 41
Questionnaires			
VAS, rest (AU)	0.9 ± 1.7	0.6 ± 1.4	1.2* ± 2.0
VAS, activity (AU)	2.5 ± 2.6	1.9* ± 2.3	2.2 ± 2.6
VAS (after 6MWT))	1.1 ± 1.9	1.1 ± 1.9	1.6 ± 2.5
IPAQ (AU)	2867 ± 2650	2630 ± 1800	2716 ± 2232

Body composition was measured by DXA scan on each of the three visits: baseline, after 2 months of statin discontinuation, and after 2 months of statin re-introduction

The visual analog scale (VAS) used was a physical Wong–Baker scale converted to a numerical value of 0.0–10.0. International Physical Activity Questionnaire–Short Form (IPAQ) questionnaire reveals a score indicating the participant’s level of activity in the week leading up to the test—a higher score indicating a higher level of activeness

Muscle capacity tests: CST (chair stand test) was converted to power (W) by method from Alcazar et al. [35]. *6MWT*, 6-min walking test

^*^Denotes different from baseline, *P* < 0.05

### Muscle performance

6MWT increased after discontinuation (from 544 ± 78 to 556 ± 80 m, *P* < 0.05) and remained increased after re-introduction (563 ± 90 m, *P* < 0.05). The same pattern was seen in CST (15.7 ± 4.3 to 16.3 ± 4 times, *P* < 0.05 after discontinuation, 16.4 ± 4.9, *P* < 0.05 after re-introduction) and QMT. HGS was unchanged throughout the trial (Table 3).

### Physical activity

The general physical activity of the participants, estimated by IPAQ score, was not altered throughout the study (Table 3).

There was no significant correlation between the participants’ daily activities (with IPAQ) and their myalgia score (VAS; data not shown).

When dividing the participants into four groups based on IPAQ score (a higher score means more physical activity during the last week), there was a non-significant tendency that the most active group experienced less myalgia than the other three groups (data not shown).

Besides myalgia measured by VAS, participants were asked if they were experiencing side effects. There was no significant crossover between the self-reported side effects and VAS or self-reported side effects and muscle tests.

### Myalgia

**Fig. 3:**
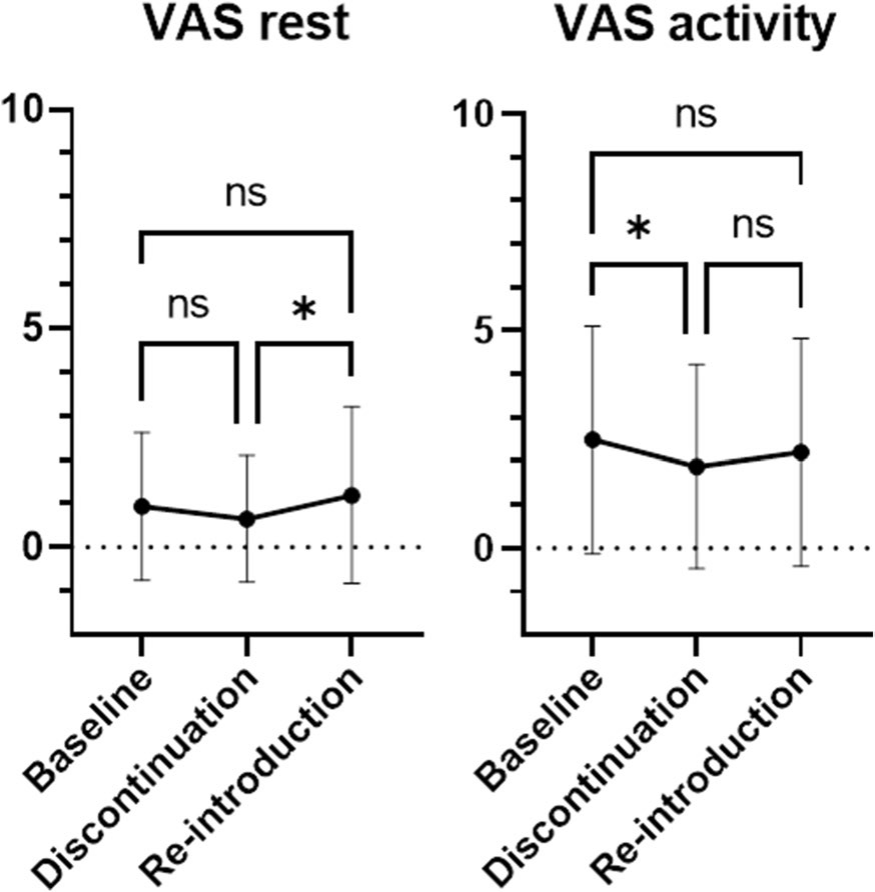
Myalgia based on visual analog scale (VAS) scores. Participants were asked to rate the myalgia in their arms and legs during the 2 weeks leading up to the examination. The scores were obtained by the use of a physical Wong–Baker scale converted to a numerical value of 0.0–10.0. One rating was given for myalgia experienced during rest and one for myalgia during activity. Data are shown as mean ± SD. * denotes *P* < 0.05

Myalgia at rest and during physical activity was estimated by VAS. A significant decrease in myalgia during physical activity was observed after the discontinuation of statins (*P* = 0.0252). Furthermore, the re-introduction of statins resulted in a significant increase in myalgia at rest (*P* = 0.0128; Fig. 3).

There was no relationship between the dosage of statins and the VAS response (data not shown).

### Blood cholesterol

**Fig. 4:**
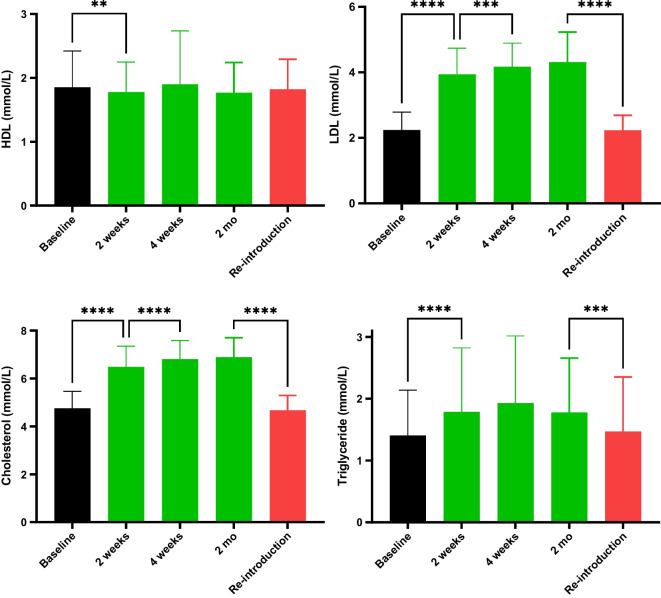
Total cholesterol, lipoproteins LDL-C and HDL-C, and triglycerides were measured during the study. One blood sample was taken at baseline (after having taken a statin for 1 + years). After discontinuation, plasma total cholesterol and lipoproteins were measured after 2 weeks, 4 weeks, and 2 months. Whereafter the statin was re-introduced and the last blood sample was measured after 2 months of statin re-introduction. Data are shown as mean ± SD. *, **, ***, and **** denotes *P* < 0.05, *P* < 0.01, *P* < 0.001, and *P* < 0.0001, respectively

A secondary outcome of the study was to delineate the effect of statin discontinuation on plasma cholesterol and lipoproteins. After 2 weeks of discontinuation, total cholesterol and LDL-C increased from 4.8 ± 0.7 to 6.5 ± 0.9 and 2.2 ± 0.5 to 3.9 ± 0.8 mM, respectively, and remained elevated until the re-introduction of statins (Fig. 4).

When comparing the low, medium, and high dosages of statin treatment, the baseline levels of total cholesterol and LDL-C were lowest for the high dosages of statin. At 2 months of discontinuation, the levels of total cholesterol and LDL-C had risen to approximately the same level for all three dosage groups.

Triglycerides followed the same pattern as total cholesterol and LDL-C, whereas high-density lipoprotein cholesterol was largely unaffected by statins (Fig. 4).

Besides total cholesterol, lipoproteins, and triglycerides, the remaining biochemical analysis showed no sign of changes through the discontinuation and re-introduction of statins.

## Discussion

### Muscle performance

This study is, to our knowledge, the first of its kind to demonstrate a connection between statin discontinuation and improved muscle performance. All functional muscle capacity tests (CST and 6MWT) showed a significant improvement after discontinuing statins, and this improvement continued for 2 months after the re-introduction of statins. We hypothesized that muscle performance would improve with the discontinuation of statin treatment, followed by a reduction of muscle performance after the re-introduction of statins. However, the improvement in muscle performance obtained with the statin discontinuation continued even after the statin treatment was re-introduced. These results may be explained by a learning effect, i.e., the participants performed better at their subsequent tests after the baseline tests and hence performed better and better. However, the participants did not have access to their previous scores and there was a minimum of 2 months between each test—which according to most studies on the learning effect is too long a period for a possible learning effect to occur [39, 40].

The results of the functional muscle capacity tests may be explained by statin-related muscle impact. This could either be by a direct statin-related muscle decomposition not yet understood or by subclinical myalgia causing less muscle activity and hence less muscle capacity over a longer period. While statin-induced myalgia typically occurs within 4–6 weeks of starting therapy, it may still occur after years of treatment [13], and it is possible that subclinical myalgia did not appear within the 2 months of re-introduction. In both of these scenarios, it seems that we see the results of several years of statin-related loss of muscle performance being lessened and that 2 months of statin-free exercise is not outdone by only 2 months of statin re-introduction.

The present study showed no significant changes in lean mass and fat tissue, as determined by DXA scanning. Although the participant’s functional muscle capacity (6MWT and CST) increased with the discontinuation of statin treatment, this did not translate into an increase in daily physical activity (IPAQ scores), and consequently, no increase in, for example, lean mass could be detected with the discontinuation of statins.

A recent study has shown a possible link between more intensive statin regimens and myalgia, making it more likely also to find a link between weakened muscle performance and larger statin dosages [41]. However, when comparing the different dosages of statins in the present study (Table 2, low, medium, and high dosages), we found no significant differences in changes in VAS, CST, 6MWT, or HGS measures between the different dosages. This could be caused by the separation of the dosage groups, hence giving less power to the statistics in this part of the study.

### Myalgia

Myalgia has previously been reported as a side effect of statin treatment in multiple studies [13, 23, 30, 33]. This was confirmed by the present study, which found a decrease in myalgia due to the discontinuation of statins for 2 months. A significant reduction in myalgia was experienced during activity without statin treatment, and participants experienced more myalgia at rest after the re-introduction of statins. Other studies have shown that muscular side effects are often most prominent during the first months of initiation [33, 42].

Earlier studies have indicated that there is no significant decrease in muscle function with statin usage. Among these are a major cross-sectional study [43] and a double-blinded study [33]. These studies did however not focus on older patients (65 + years) but found that increased age amplified the risk of myalgia compared with younger test subjects [33].

A recent meta-analysis by Reith et al. [41] compared the muscular side effects of different statin dosages. The study determined that higher dosage regiments yielded a higher relative risk of myalgia. Our sample sizes are significantly smaller, and we could not confirm this relationship.

By using the VAS score, the present study confirms the presence of myalgia with statin treatment and the reduction of this with discontinuation of statin treatment. Mild myalgia was present in > 20% of participants (VAS > 2), but neither this group nor the totality of participants showed signs of loss of muscle mass (estimated by lean mass) or reduced daily activity. Thus, myalgia and muscle symptoms were not associated with a decrease in physical activity. This lack of correlation between myalgia and muscle performance is supported by Morville et al. [30].

### Blood cholesterol

This study also examined the relationship between statin discontinuation and blood lipid concentrations. This was done to ensure compliance with the study protocol and as a way of examining the median blood lipid concentrations in patients who had been taking statins for many years.

The data showed that plasma total cholesterol and lipoproteins (specifically LDL-C) had a quick and significant response to the discontinuation and re-introduction of statins.

The total cholesterol concentrations increased significantly with the intervention and decreased significantly with the re-introduction of statins. While the correlation was unsurprising, the clear and significant effect on all participants, even those whose dosage had not been altered for 10 + years, was astounding.

With stricter cholesterol guidelines and an aging population, it has been estimated that more than 90% of individuals between 65 and 75 years would meet the criteria for considering statin treatment according to the American College of Cardiology/American Heart Association (AHA) guidelines [44, 45]. The goal of LDL-C < 1.8 mmol/L for patients of moderate risk is the same between SCORE and AHA guidelines and has previously included most older people because age is one of the most significant risk factors. This is perhaps the reason that most guidelines do not include risk stratification for people older than 79 years. However, the recent changes in the ESC recommendations with age-adjusted intervention recommendations could change the prescription habits in Europe in the near future [19]. The pendulum could also swing the other way as a large cohort study has recently found that LDL-C levels are even more important for the oldest patients compared to younger patients when determining the risk of cardiovascular events [25]. This means that even the oldest patients (> 90 years) may be recommended statins as primary prevention in years to come.

When comparing the different statin dosages, it was surprising to find that plasma total cholesterol and LDL-C rose to around the same levels for all participants independent of which statin dosage they were treated with before the discontinuation. The higher dosage of statin did lower the levels of total cholesterol and LDL-C more at baseline and after re-introduction than the lower dosages. We would, however, have expected the high-dosage participants to have higher plasma cholesterol when untreated. This shows that overall the participants were appropriately treated by their general practitioner and that there were large inter-subject differences in their response to statins.

### Limitations

The participants knew that we examined possible side effects of statins, and they may have been more susceptible to and focused on muscle soreness during the trial. However, the participants could not possibly remember their scores from time to time, and these were not given to them until after the trial had ended for each individual.

Familiarization with the tests could have improved test performances. This is possible when looking at most of the muscle function tests that showed signs of general improvement over time. This has been taken into account with the single-subject experimental design and is the main reason for the third control test after the re-introduction of statin treatment.

The test–retest validity of 6MWT and CST is generally considered high, with intra-class correlation coefficients between 0.94 and 0.99, and between 0.84 and 0.92, respectively [46–48]. Some studies examining the retest validity of 6MWT and CST showed a learning effect from the first to the second test, whereas others found no significant change in distance walked when retesting the same day [46–48]. The magnitude of the documented learning effects is around 2% for the 6MWT on trials performed the same day and is highly reliable over 1 year [46].

## Conclusion and perspectives

The effect of statin treatment on skeletal muscle in older persons was observed in several different ways in this study: lean mass (by DXA), muscle performance (by functional muscle capacity and muscle strength), and sensation (by VAS and IPAQ). Two months of discontinuation of statin treatment elicited significant increases in plasma cholesterol and muscle performance. The increases in plasma total cholesterol and LDL-C were quickly reversed by re-introduction, whereas improvements in muscle performance were more permanent.

The study period of 4 months may be too short a period for showing the temporary statin-related loss of strength. The problem with extrapolating the study with a longer discontinuation period is the fact that in this study group of 65 + in primary prevention, the ethical risk of discontinuation cannot be overlooked. However, there are an increasing number of randomized clinical trials examining the effects and side effects of statins—including a study of 20,000 healthy participants above the age of 75 years, comparing atorvastatin (40 mg) vs. placebo. However, the publication of data is not expected before 2027 [49]. A similar study is underway in Australia with the STAREE trial examining the events and adverse effects of atorvastatin vs placebo in approximately 10,000 healthy participants [50].

The results from the present study, especially data on myalgia and muscle performance, are considered relevant for clinical practice and public health when it comes to the decision-making process for initiating statin treatment in primary prevention in older people. This should give clinical personnel an even better opportunity to balance the many pros and not so many cons of statin treatment.

## Data Availability

The data that support the findings of this study are available from the corresponding author upon reasonable request.
